# The influences of daily experiences of awe on stress, somatic health, and well-being: a longitudinal study during COVID-19

**DOI:** 10.1038/s41598-023-35200-w

**Published:** 2023-06-08

**Authors:** María Monroy, Özge Uğurlu, Felicia Zerwas, Rebecca Corona, Dacher Keltner, Jake Eagle, Michael Amster

**Affiliations:** 1 Department of Psychology, University of California, Berkeley, 2121 Berkeley Way, MC #5050, Berkeley, CA 94720-5050 USA; 2 College of Osteopathic Medicine, Touro University California, Vallejo, USA; 3 Department of Psychiatry and Behavioral Sciences, University of California, San Francisco, San Francisco, USA; 4 Independent Scientist, Hawi, Hawaii USA

**Keywords:** Psychology, Signs and symptoms

## Abstract

In the present work, we used daily diary methodology to investigate the influence of awe on stress, somatic health (e.g., pain symptoms), and well-being during the COVID-19 pandemic in 2020. We recruited a sample of community adults (*N* = 269) and a sample of healthcare professionals (*N* = 145) in the United States. Across both samples, we found that awe and well-being increased, and stress and somatic health symptoms decreased over the 22-day diary period. In daily level analyses, we found that the more daily awe people experienced, the less stress, less somatic health symptoms, and greater well-being they felt. Daily experiences of awe can benefit individuals during times of acute and chronic stress—such as the COVID-19 pandemic.

## Introduction

Awe is an emotion long theorized to benefit the mind and body^1–4^. This thesis is supported in a recent review of how the awe felt across five domains—nature, music, collective movement, psychedelics, and spiritual contemplation—benefits mental and physical health^5^. In the present work, we build on this literature by examining the influence of daily experiences of awe on stress, somatic health symptoms, and well-being during a time of global trauma, the COVID-19 pandemic.

### Stress and well-being

Stress—feeling under-resourced in meeting present challenges—is a central focus in studies of health and well-being^6, 7^. Stress, in particular the prolonged experience of stress (i.e., chronic stress), has well documented influences upon: elevated anxiety, depression, risk of disease; diminished physical health, for example as indexed by inflammation or telomere length; and reduced well-being^8–12^.

As we write in 2022, the COVID-19 pandemic has proven to be a profound source of chronic stress across ages, cultures and ethnicities. Over the course of the pandemic, there has been a 25% rise in prevalence of anxiety and major depression worldwide^13^. This is in line with other reports showing that during the pandemic people in the United States reported higher levels of psychological distress (e.g., anxiety, depression, sleeplessness)^14^ and higher stress as compared to previous years^15^. These burdens of chronic stress were amplified for healthcare professionals, on the front line of the pandemic, who were helping people fight infection and encountering upwards of one million deaths. Studies find that there was an increase in stress, depression, anxiety, and physical burnout for healthcare workers^16–18^. In light of these findings, we sought to examine whether daily experiences of awe would reduce levels of stress in healthcare providers and community adults during the COVID-19 pandemic.

### The awe, stress, well-being dynamic

A central tenet to emerge from the study of stress is that positive emotions can counteract the detrimental effects of stressful experiences^19–22^. This has been documented in in-depth studies of positive emotions: laughter and amusement were associated with improved psychological functioning during bereavement^23^; and overall positive emotions buffered people against depression and increased well-being in the aftermath of a traumatic event, such as a terrorist attack^24^. This has also emerged as a central theme in recent meta-analyses and empirical reviews that suggest that boosts in positive emotion reduce stress and enhance physical health and well-being^21, 22^. Cultivating positive emotions is a resilience factor.

Within the new science of awe, studies have begun to uncover how brief experiences of this emotion bring about beneficial effects on mental and physical health^5^. Awe is an emotion conceptualized as primarily positive, and it’s elicited by vast stimuli that do not fit into existing mental schemas^1^. Awe is often experienced through encounters with other people’s courage and kindness, nature, collective gatherings (dance, rituals, and ceremonies), music, visual art, religious and spiritual practice, epiphanies, and birth and death^1, 2, 5^. Empirical studies find experiences of awe to be associated with increased vagal tone^25^, reduced activation of the sympathetic nervous system^26, 27^, and lower inflammation, as indexed by the biomarker interleukin-6^28^.

Germane to the current research, studies find that awe is associated with lower levels of stress related measures. For example, studies found that dispositions to experience awe and daily reports of awe were associated with lower subjective stress^2^. Notably, these effects held when controlling for experiences of other positive emotions. In laboratory studies, inductions of awe reduced physiological reactions to stress (i.e., sympathetic autonomic arousal)^2^. Awe experiences in nature were associated with decreased rumination^29^, and reductions of stress and PTSD symptoms in samples of adolescents from under-resourced inner-city schools and combat veterans.^3^

Evidence also points to the beneficial effects of awe on well-being outcomes. In studies across diverse methodologies—in lab, daily diary, and field studies—awe predicted greater satisfaction with life^2, 3, 25, 30^ and greater overall well-being^3^. This effect of awe on well-being, which is robust and replicable, suggests that awe is a strong driver of human flourishing. In the present investigation, we expand on this literature by examining the effects of awe on somatic health symptoms, subjective stress, and well-being during the COVID-19 pandemic in 2020 in samples of community adults and healthcare professionals.

### The current research

In the present investigation, we examine how daily awe experiences might influence somatic health symptoms, subjective stress, and well-being in a sample of community participants and healthcare professionals during the peak of COVID-19 pandemic. Data collection took place during June of 2020, a time when the world was full of uncertainty: COVID-19 was at its peak around the world, people were indefinitely quarantined at home, infections and deaths were increasing, the vaccine was not yet available, and hospitals in the USA (and all over the world) were at maximum capacity. COVID-19 had detrimental effects on people’s physical and psychological health, as evident in a 25% rise worldwide in prevalence of depression and anxiety^13^. The COVID-19 pandemic was and is still clearly a time of stress and trauma for all individuals, which is likely amplified for healthcare workers as they continue to be at the forefront of the pandemic. Across two samples, using a daily diary methodology, we examined the beneficial effects of daily experiences of awe. We hypothesized that daily experiences of awe would be associated with (H1) lower subjective stress, (H2) lower somatic health symptoms, and (H3) greater well-being.

## Method

### Participants

The current study consists of two samples. Sample 1 consisted of 386 community adults and was reduced to a final sample size of 269 after data cleaning procedures (e.g., late entries, less than five entries; see preliminary analyses section). Community adults were recruited from the NorthBay Healthcare system and the broader community in California and across the USA. Sample 2 consisted of 227 healthcare professionals and was reduced to a final sample size of 145 after data cleaning procedures (see preliminary analyses section). Healthcare professionals were recruited from Northern California healthcare centers and other health centers across the USA and included individuals who were currently working or had worked in healthcare (e.g., physicians, physician assistants, therapists, nurses, and administrative staff). The sample sizes are comparable to those of previous studies examining daily experiences of awe and other positive emotions^2, 3^ and the repeated measures design of our study provides greater statistical power (see online supplemental materials for power analyses). See Table 1 for sample descriptives.

**Table 1:** Sample descriptives.

	Community (%)	Healthcare (%)
Gender		
Female	85.5	83.4
Male	11.5	14
Other	3	2.8
Age		
19–29	2.2	1.4
30–39	10.4	17.9
40–49	23.8	29.7
50–59	20.4	26.9
60–69	24.9	19.3
70 +	16	3.4
Ethnicity		
White American	80.7	72.4
Black/African American	1	1.4
Latinx American	3.7	2.1
East Asian/Asian American	3.7	8.3
Other	5.9	6.9
Mixed ethnicity	4.8	8.3

Six participants for Sample 1 and two for Sample 2 did not provide demographic information.

### Procedure

Participants were recruited during May 2020. NorthBay Healthcare, a non-profit mid-sized healthcare system serving Solano County, CA., reached out to thousands of patients and 300 healthcare professionals with HIPAA compliant email, press announcements, and online social media marketing about enrollment in the study. For the community sample, friends and family of patients were also invited to enroll in the study. For the healthcare sample, colleagues from other institutions were also invited to enroll in the study. All interested participants provided their contact information via a Qualtrics online survey.

Participants were first instructed on the structure of the study via email, and they received a link to the initial online survey. In the initial survey, they provided informed consent, completed demographic questions, personality measures, and baseline well-being. Two days later, participants took part in a 60-min online zoom session, consisting of an overview of the study and the science of awe, that began at 12:15 pm PDT for the healthcare sample and at 7 pm PDT for the community sample (see supplemental materials). Then, the daily diary began. Participants were sent a link to the online diary every day at 4 pm for 22 consecutive days. Each diary began with Likert-type questions that prompted participants to report on their emotions, thoughts, and experiences that day. At the end of the diary, participants were asked to write a short narrative about their favorite moment of awe of that day. A day after the final diary entry, participants completed follow-up well-being measures. All participants provided informed consent and all aspects of the study design and procedure were approved by the NorthBay Healthcare Institutional Review Board (NBH IRB). The study was conducted in accordance with guidelines and regulations of the NBH IRB and with the Declaration of Helsinki ethical standards.

### Measures

#### Initial measures

In the initial questionnaire, participants provided demographic information, personality measures (e.g., trait awe), and baseline well-being measures. Trait awe was assessed via the awe subscale of the dispositional positive emotion scale (DPES)^31^. Participants responded to six items indicating their agreement with statements such as “I often feel awe,” on a scale from 1 (*strongly disagree*) to 7 (*strongly agree*). Baseline well-being was measured using the Mental Health Continuum Short Form (MHC-SF)^32, 33^. Participants responded to fourteen items indicating the frequency of how they felt during the past two weeks (e.g., *satisfied with life*, *that [they] had something important to contribute to society, that [they] had experiences that challenged [them] to grow and become a better person*), on a scale ranging from 1 (*never*) to 6 (*every day*). See Table 2 for descriptive statistics.

**Table 2:** Descriptive statistics.

	Sample 1 (*N* = 269)		Sample 2 (*N* = 145)	
	*M* (*SE*)	ω_b (_ω_w)_	*M* (*SE*)	ω_b (_ω_w)_
Initial Measures				
DPES-Awe	4.99 (.07)	.84	4.98 (.10)	.88
Well-being (T1)	3.90 (.06)	.92	4.16 (.08)	.92
Daily Measures_*across diary*_				
Awe	2.75 (.04)		2.81 (.06)	
Positive Emotionality	3.11 (.04)	.94 (.79)	3.20 (.06)	.94 (.79)
Amusement	2.63 (.04)		2.71 (.06)	
Compassion	3.46 (.05)		3.57 (.06)	
Contentment	2.90 (.04)		2.99 (.06)	
Gratitude	3.44 (.05)		3.43 (.07)	
Love	3.44 (.05)		3.52 (.07)	
Pride	2.77 (.04)		3.00 (.07)	
Subjective Stress	2.06 (.05)		2.05 (.06)	
Somatic Health Symptoms	1.53 (.03)	.85 (.55)	1.46 (.03)	.87 (.61)
Stomach problems	1.41 (.03)		1.41 (.04)	
Back pain	1.56 (.05)		1.44 (.05)	
Neck pain	1.52 (.04)		1.46 (.06)	
Arm, legs, joint pain	1.71 (.06)		1.49 (.05)	
Headaches	1.35 (.03)		1.29 (.04)	
Chest pain, shortness of breath	1.08 (.01)		1.10 (.02)	
Dizziness	1.14 (.02)		1.12 (.03)	
Fatigue	2.15 (.06)		2.08 (.07)	
Sleeping problems	1.87 (.05)		1.76 (.07)	
Well-being	3.09 (.05)	.94 (.79)	3.34 (.07)	.96 (.81)
Post-Diary Measures				
Well-being (T2)	4.47 (.06)	.93	4.65 (.08)	.94

Sample sizes for initial measures were 264 for Sample 1 and 143 for Sample 2, and for the post-diary measures were 212 and 113 respectively. Well-being (T1) denotes baseline well-being. DPES-Awe denotes dispositional awe. Well-being (T2) denotes well-being post-diary. Omegas denote estimates of reliability between-person ω_b_ and within-person ω_w_ (for daily measures only).

#### Daily diary measures

In each diary entry, participants were asked about the following thoughts and feelings they had that day. See Table 2 for descriptive statistics.

##### Awe

Guided by previous studies measuring emotional experiences^3, 34, 35^ and recent studies of positive emotion^36, 37^, awe was assessed with a single item composed of a synonym cluster (*awe/amazed/wonder*), in which participants rated how much awe they experienced that day on a scale from 1 (*none at all*) to 5 (*a great deal*).

##### Positive emotions

Positive emotions were assessed with single items composed of synonym clusters^3, 34, 35, 38^, in which participants rated how much of each of six positive emotions they experienced each day on a scale from 1 (*none at all*) to 5 (*a great deal)*: Amusement (*amused/having fun/laughing*), Compassion (*compassionate/sympathetic/concern for others*), Contentment (*content/relaxed/peaceful*), Gratitude *(grateful/appreciative/thankful*), Love *(love/affection/warmth*), and Pride *(proud/sense of accomplishment/successful*). To assess daily positive emotionality, all six items were combined into a composite.

##### Subjective stress

Subjective stress was measured with a single item composed of a synonym cluster (stressed/overburdened/pressured)^2^ on a scale from 1 (*none at all*) to 5 (*a great deal*).

##### Somatic health symptoms

Somatic health symptoms were measured using the 8-item Somatic Symptom Scale^39^ and an additional face-valid item assessing neck pain. Participants rated whether they had any of nine symptoms that day on a scale from 1 (*not at all*) to 5 (*a great deal*): *Stomach or bowel problems*; *Back pain*; *Neck pain*; *Pain in your arms legs, or joints*; *Headaches*; *Chest pain or shortness of breath*; *Dizziness; Feeling tired or having low energy*; *Trouble sleeping*. To assess daily somatic health symptoms, all nine items were combined into a composite.

##### Well-being

Daily well-being was assessed with a modified short five item version of the MHC-SF^32, 33^. Participants indicated their agreement on a scale from 1 (*does not describe me*) to 5 (*describes me extremely well*): *I felt satisfied with life, I felt that my life has a sense of direction or meaning to it, I felt good at managing the responsibilities of my daily life, I felt that I belonged to a community (like a social group, your school, or your neighborhood), I enjoyed life.* To assess daily well-being, all five items were combined into a composite.

### Post-diary measures

Well-being, post-diary (T2), was measured using the MHC-SF^32, 33^, as in the initial measures. The MHC-SF is a well-rounded measure of well-being, including emotional, psychological, and social well-being^32, 33^. Participants responded to fourteen items indicating the frequency of how they felt during the past 21 days on a scale from 1 (*never*) to 6 (*every day*). See Table 2 for descriptive statistics.

### Data analytic plan

All statistical analyses were performed using RStudio in the R programming environment (version 4.1.2). Our preliminary analyses included examination of missing data and data exclusion based on the planned specified criteria.

For our primary analyses, with daily-level variables, we used Hierarchical Linear Modeling. The repeated measures design resulted in a two‐level hierarchical structure with daily diaries nested in participants, and we included random intercepts and random slopes for participants. We fitted our models using the *lme4* and *lmerTest* packages (versions 1.1-27.1 and 3.1-3); the degrees of freedom (*df*) and p-values were calculated using Satterthwaite’s method^40^, which yields *df* that are somewhere between the number of total observations, the individuals, and days depending on the relative variance explained by each factor. This explains the variability in *df* from model to model.

One strength of a multilevel-modeling approach is that it allows for the examination of day-level effects (i.e., within-person effects), where we can test whether daily changes in one variable are associated with daily changes in another variable. For example, on days in which people report high levels of awe, do they also report greater well-being? To examine day-to-day effects, we person-centered daily variables, where outcomes represent changes in a variable from that person’s own average.

### Ethical approval

All aspects of the studies design and procedures were approved by the NorthBay Healthcare Institutional Review Board.

### Informed consent

All participants provided informed consent.

## Results

### Sample 1: Daily awe in community adults

#### Preliminary analysis

Of the total sample (*N* = 386), the final sample for analyses consisted of participants with at least five entries over the course of the 22-day period submitted between 4 pm and 6 am. That is, given our analytic plan and the complexity of some of the models, we only included participants with five or more diary entries. To ensure each diary entry was reflective of a specific day, entries submitted after 6am the following day were also excluded from the final sample used for analyses. International participants, who were not residing in the USA, were also excluded given the large gaps in time zones. Further cleaning details can be found in the online supplemental material. The remaining 269 participants (70% of total *N*) submitted a total of 4052 diary entries out of the possible 5918 (68%) during the diary period.

### Daily level analyses

#### Trajectories of awe, stress, somatic health, and well-being

Before testing our main hypotheses and in exploratory form, we examined the effect of time using 2-level hierarchical linear models (HLMs) to determine whether time should be accounted for in the main models. First, we examined the trajectory of awe, to test if awe increased over time. We found that awe increased over the 22-day period (*β* = 0.14, 95% CI [0.11, 0.17], *p* < 0.001). This effect remained significant (*p* < 0.001) when controlling for the predisposition to experience awe (i.e., trait awe). This suggests that daily awe increased over time for everyone, regardless of awe predisposition. We next examined the trajectories of our other variables of interest—subjective stress, somatic health symptoms, and well-being. In separate models, we found that subjective stress (*β* =− 0.12, 95% CI [− 0.15, − 0.09], *p* < 0.001) and somatic health symptoms (*β* =− 0.09, 95% CI [− 0.12, − 0.07], *p* < 0.001) decreased, and well-being increased over the 22-day-period (*β* = 0.03, 95% CI [0.01, 0.06], *p* = 0.013). The increase in well-being held (*p* = 0.016) when controlling for baseline well-being. These findings suggest that as time progressed during the diary period, people experienced more awe, less stress, less somatic health symptoms, and greater well-being.

#### Influences of awe upon stress, somatic health, and well-being

Given the significant effect of time on awe, stress, somatic health, and well-being, we control for time in the following models. To test our first Hypothesis (H1), we examined the relationship between daily awe and daily subjective stress in a 2-level HLM. As predicted, we found that on the days that people reported experiencing more awe than typical, they reported less stress (*β* =− 0.20, *p* < 0.001). Similarly, and consistent with Hypothesis 2 (H2), on the days that people reported experiencing more awe than typical, they reported less somatic health symptoms (*β* =− 0.08, *p* < 0.001). As predicted in Hypothesis 3 (H3), on the days that people reported experiencing more awe than typical, they reported greater well-being (*β* = 0.22, *p* < 0.001). See Table 3 for model statistics.

**Table 3:** Daily level analyses.

Predictors	Subjective stress					Somatic health symptoms					Well-being				
	*β*	*CI*	*t*	*df*	*p*	*β*	*CI*	*t*	*df*	*p*	*β*	*CI*	*t*	*df*	*p*
Daily Awe	− 0.20	− 0.22 to − 0.17	− 13.56	598.24	< 0.001	− 0.08	− 0.10 to −0.06	− 7.74	241.44	< 0.001	0.22	0.20 to 0.24	20.49	240.92	< 0.001
Time	− 0.09	− 0.12 to − 0.05	− 5.52	236.61	< 0.001	− 0.08	− 0.10 to − 0.06	− 6.70	204.53	< 0.001	− 0.01	− 0.03 to 0.01	− 0.72	216.39	0.470
Random effects															
σ^2^	0.48					0.05					0.19				
τ_00_	0.63 _ID_					0.22 _ID_					0.70 _ID_				
τ_11_	0.05 _ID.Awe_					0.00 _ID.Awe_					0.02 _ID.Awe_				
	0.00 _ID.Time_					0.00 _ID.Time_					0.00 _ID.Time_				
ρ_01_	− 0.47					− 0.20					− 0.21				
	− 0.30					− 0.41					− 0.26				
ICC	0.57					0.80					0.78				
N	269 _ID_					269 _ID_					269 _ID_				
Observations	4052					4041					4039				
Marginal R^2^/ Conditional R^2^	0.052/0.594					0.014/0.804					0.050/0.788				

The random effects notation indicates the following: σ^2^ denotes within-person variance (level 1); τ_00_ indicates between-person variance of ID (i.e., subjects; level 2); τ_11_ denotes between-person variance of Daily Awe and Time (i.e., day); ρ_01_ indicates the correlation between random intercepts and slopes at level 2. ICC denotes the intraclass correlation. Marginal R^2^ indicates variance explained by the fixed effects and conditional R^2^ indicates variance explained by both fixed and random effects, for each respective model. All estimates are standardized.

We then examined if our models would hold when controlling for the disposition to experience awe (for H1 and H2) and baseline well-being (for H3), two crucial analyses for ascertaining the robustness of the daily awe effects. Our models held when controlling for trait awe for subjective stress (H1; *β* =− 0.20, 95% CI [− 0.23, − 0.17], *p* < 0.001) and somatic health symptoms (H2; *β* =− 0.08, 95% CI [− 0.10, − 0.06], *p* < 0.001). For H3, awe predicted greater well-being (*β* = 0.22, 95% CI [0.20, 0.24], *p* < 0.001), when controlling for both trait awe and baseline well-being.

Next, given the beneficial effects of overall positive emotional experiences^20, 41^, we examined whether the effects were unique to awe and not an overall effect of positive emotionality. To do so, in addition to the aforementioned control variables (i.e., time, trait awe, and baseline well-being), we added daily positive emotions to our models. We found that the effects of awe remained significant when controlling for daily positive emotionality for subjective stress (*β* =− 0.08, 95% CI [− 0.10, − 0.05], *p* < 0.001), somatic health symptoms (*β* =− 0.03, 95% CI [− 0.05, − 0.01], *p* = 0.003), and well-being (*β* = 0.05, 95% CI [0.04, 0.07], *p* < 0.001). These findings suggest that on days when people experienced more awe, beyond general positive emotion, stress and somatic health symptoms decreased and well-being increased.

### T2 Well-being analyses

Having established our daily level effects, we next tested H3 with our T2 well-being measure, assessed after the daily diary, in a between-person analysis. We found that awe across the 22 days predicted greater well-being after the diary (*β* = 0.46, *p* < 0.001), and this effect held when controlling for baseline well-being (see Table 4, Model 2). This suggests that the awe experienced across 22 days had a unique effect on T2 well-being, above and beyond baseline levels of well-being. We then added dispositional awe as a control variable and found that the effect of daily awe remained significant (*β* = 0.26, *p* < 0.001).

**Table 4:** Between-person analyses with T2 well-being as the outcome variable.

Predictors	Well-being															
	Model 1				Model 2				Model 3				Model 4			
	*β*	*CI*	*t*	*p*	*β*	*CI*	*t*	*p*	*β*	*CI*	*t*	*p*	*β*	*CI*	*t*	*p*
Awe	0.46	0.33 to 0.58	7.42	< 0.001	0.23	0.13 to 0.34	4.39	< 0.001	0.26	0.15 to 0.37	4.51	< 0.001	0.07	− 0.09 to 0.22	0.86	0.389
Well-being (T1)					0.58	0.48 to 0.69	10.95	< 0.001	0.61	0.49 to 0.72	10.76	< 0.001	0.53	0.42 to 0.65	9.15	< 0.001
DPES- Awe									− 0.07	− 0.19 to 0.05	− 1.16	0.247	− 0.10	− 0.21 to 0.02	− 1.65	0.101
Positive Emotions													0.30	0.13 to 0.46	3.48	0.001
Observations	212				208				208				208			
R^2^/R^2^ adjusted	0.208/0.204				0.503/0.498				0.506/0.499				0.534/0.524			

Well-being (T1) denotes baseline well-being. DPES-Awe denotes dispositional awe. All estimates are standardized.

When adding self-reported positive emotionality to a model of awe predicting T2 well-being while controlling for baseline well-being and dispositional awe, we found that positive emotions accounted for the effect of awe on well-being (see Table 4, Model 4). This suggests that, in a between-person analysis, the effect of awe on well-being is not unique to awe and might be an effect of positive emotionality experienced across the 22 days.

### Sample 2: Daily awe in healthcare professionals

#### Preliminary analysis

From the total sample (*N* = 227), the final sample for analyses consisted of participants with at least five entries submitted between 4 pm and 6 am: participants who completed less than five diary entries and entries submitted after 6 am the following day were excluded from the final sample. Participants who were not residing in the USA, were also excluded (see supplemental materials). The remaining 145 participants (64% of total *N*) submitted a total of 2056 diary entries out of the possible 3190 (64%) during the diary period. Of those 145, 129 participants were active healthcare professionals. The findings reported below include the healthcare professionals that were not currently employed; results were identical when excluding this subset of participants.

### Daily level analyses

#### Trajectories of awe, stress, somatic health, and well-being

As in Sample 1, we first examined the effect of time on variables of interest. We found that awe increased over time (*β* = 0.13, 95% CI [0.09, 0.18], *p* < 0.001), and this effect remained significant (*p* < 0.001) when controlling for the predisposition to experience awe. Subjective stress (*β* =− 0.12, 95% CI [− 0.16, − 0.08], *p* < 0.001) and somatic health symptoms (*β* =− 0.13, 95% CI [− 0.16, − 0.09], *p* < 0.001) decreased over time. Well-being increased over the diary-period (*β* = 0.05, 95% CI [0.01, 0.08], *p* = 0.007), and this effect held (*p* = 0.009) when controlling for baseline well-being. These findings suggest that as time progressed during the diary period, in the peak of the COVID-19 pandemic, healthcare professionals experienced more awe, less stress, less somatic health symptoms, and greater well-being.

#### Influences of awe upon stress, somatic health, and well-being

In a similar manner as in Sample 1, and given the significant effect of time on awe, stress, somatic health, and well-being, in the following models we control for time. Replicating the findings of Sample 1, we found that on the days that healthcare professionals reported experiencing more awe than typical, they also reported less stress (*β* =− 0.20, *p* < 0.001; H1), less somatic health symptoms (*β* =− 0.09, *p* < 0.001; H2), and greater well-being (*β* = 0.21, *p* < 0.001; H3). See Table 5.

**Table 5:** Daily level analyses.

Predictors	Subjective Stress					Somatic Health Symptoms					Well-being				
	*β*	*CI*	*t*	*df*	*p*	*β*	*CI*	*t*	*df*	*p*	*β*	*CI*	*t*	*df*	*p*
Daily Awe	− 0.20	− 0.23 to − 0.16	− 10.13	480.24	< 0.001	− 0.09	− 0.12 to − 0.06	− 5.92	124.35	< 0.001	0.21	0.18 to 0.24	14.38	129.59	< 0.001
Time	− 0.09	− 0.13 to − 0.04	− 4.08	113.81	< 0.001	− 0.11	− 0.15 to − 0.08	− 6.54	127.86	< 0.001	0.01	− 0.02 to 0.04	0.50	116.24	0.615
Random effects															
σ^2^	0.55					0.06					0.22				
τ_00_	0.70 _ID_					0.20 _ID_					0.75 _ID_				
τ_11_	0.04 _ID.Awe_					0.00 _ID.Awe_					0.02 _ID.Awe_				
	0.00 _ID.Time_					0.00 _ID.Time_					0.00 _ID.Time_				
ρ_01_	− 0.36					− 0.20					− 0.09				
	− 0.50					− 0.56					− 0.09				
ICC	0.52					0.73					0.78				
N	145 _ID_					145 _ID_					145 _ID_				
Observations	2056					2050					2049				
Marginal R^2^/ Conditional R^2^	0.054/0.550					0.023/0.735					0.045/0.790				

The random effects notation indicates the following: σ^2^ denotes within-person variance (level 1); τ_00_ indicates between-person variance of ID (i.e., subjects; level 2); τ_11_ denotes between-person variance of Daily Awe and Time (i.e., day); ρ_01_ indicates the correlation between random intercepts and slopes at level 2. ICC denotes the intraclass correlation. Marginal R^2^ indicates variance explained by the fixed effects and conditional R^2^ indicates variance explained by both fixed and random effects, for each respective model. All estimates are standardized.

Next, we tested if our models would hold when controlling for trait awe, for H1 and H2, and baseline well-being for H3. Replicating Sample 1 findings, our models held when controlling for trait awe for subjective stress (*β* =− 0.20, 95% CI [− 0.24, − 0.16], *p* < 0.001; H1) and somatic health symptoms (*β* =− 0.09, 95% CI [− 0.12, − 0.06], *p* < 0.001; H2). Consistent with H3, awe predicted greater well-being (*β* = 0.21, 95% CI [0.18, 0.24], *p* < 0.001), when controlling for both trait awe and baseline well-being.

We next examined if our models would hold when accounting for overall positive emotionality. In addition to the control variables (i.e., time, trait awe, and baseline well-being), we added daily positive emotions to our models. We found that the effects of awe remained significant when controlling for daily positive emotions for subjective stress (*β* =− 0.09, 95% CI [− 0.13, − 0.05], *p* < 0.001) and well-being (*β* = 0.05, 95% CI [0.02, 0.07], *p* < 0.001), but not for somatic health symptoms (*β* =− 0.02, 95% CI [− 0.05, 0.01], *p* = 0.31). These results suggest that awe had a unique effect on healthcare professionals’ daily subjective stress and daily well-being, beyond the effects of positive emotions, but not on somatic health symptoms.

### T2 Well-being analyses

We, lastly, tested H3 with our T2 well-being measure in a between-person analysis. We found that awe across the 22 days predicted greater T2 well-being (*β* = 0.60, *p* < 0.001), and this effect held when controlling for baseline well-being (see Table 6, Model 2). Suggesting that the awe experienced across 22 days had a unique effect on well-being assessed after the diary period, above and beyond baseline levels of well-being. We then added dispositional awe as a control variable and found that the effect of daily awe held (*β* = 0.32, *p* < 0.001). When adding positive emotions to the model, the effect of awe became marginal (*p* = 0.07), and positive emotions had no significant effect on well-being (*p* = 0.10; see Table 6, Model 4).

**Table 6:** Between-person analyses with T2 Well-being as the outcome variable.

Predictors	Well-being															
	Model 1				Model 2				Model 3				Model 4			
	*β*	*CI*	*t*	*p*	*β*	*CI*	*t*	*p*	*β*	*CI*	*t*	*p*	*β*	*CI*	*t*	*p*
Awe	0.60	0.46 to 0.75	8.00	< 0.001	0.33	0.19 to 0.46	4.85	< 0.001	0.32	0.17 to 0.47	4.27	< 0.001	0.19	− 0.02 to 0.41	1.82	0.072
Well-being (T1)					0.57	0.44 to 0.71	8.44	< 0.001	0.58	0.44 to 0.72	8.08	< 0.001	0.52	0.36 to 0.68	6.60	< 0.001
DPES-Awe									0.01	− 0.14 to 0.16	0.11	0.915	0.01	− 0.14 to 0.16	0.09	0.931
Positive Emotions													0.19	− 0.04 to 0.42	1.64	0.103
Observations	113				111				111				111			
R^2^/R^2^ adjusted	0.366/0.360				0.617/0.610				0.625/0.614				0.634/0.620			

Well-being (T1) denotes baseline well-being. DPES-Awe denotes dispositional awe. All estimates are standardized.

Taken together, these findings replicate those of Sample 1 and suggest that daily experiences of awe are associated with a reduction in subjective stress, somatic health symptoms, and improvements in well-being for healthcare workers. The effects of awe were unique to awe and not just an effect of overall positive emotions, at the day-to-day level.

Lastly, in ancillary analyses, to obtain average effect sizes across the two samples, we conducted an internal meta-analysis to examine the independent effects of awe beyond positive emotions. We found unique associations between daily awe and daily subjective stress (*β* = − 0.08, 95% *CI* [− 0.10, − 0.06]), daily somatic health (*β* = − 0.03, 95% *CI* [− 0.05, − 0.01]), and daily well-being (*β* = 0.05, 95% *CI* [0.04, 0.06]). Awe across the 22-day diary period did not uniquely predict T2 well-being (*β* = 0.11, 95% *CI* [− 0.02, 0.24]). Taken together, our findings suggest that across both samples, awe is uniquely associated with reduced stress, somatic health symptoms, and greater well-being at the daily level, but not in follow-up measures.

## Discussion

Awe is beneficial for health and well-being^4, 5^. In the present investigation, we expand on the science of awe by examining the effects of daily awe on stress, somatic health, and well-being in the midst of the COVID-19 pandemic in 2020.

Over the course of the 22-day diary period, we observed an upshift in awe and well-being, and a downshift in subjective stress and somatic health symptoms (e.g., less headaches, trouble sleeping). Critically, the increases in awe and well-being held when controlling for trait awe and baseline well-being, suggesting that these shifts might be due to daily experiences and not driven by individual baseline levels and dispositional tendencies.

In daily level analyses, we found that daily awe predicted less subjective stress (H1), less somatic health symptoms (H2), and greater well-being (H3). In other words, on days when community adults and healthcare professionals reported experiencing more awe than typical, they also felt less stressed, experienced less body pains and problems sleeping, and felt greater well-being. Critically, these effects held when controlling baseline well-being for H3, and trait awe and daily positive emotionality across all the models (H1-3).

In a between-person analysis of well-being assessed after the daily diary, we found that awe across the 22-day diary period predicted greater well-being at follow-up. This effect held when controlling for trait awe and baseline levels of well-being. When controlling for positive emotionality across the 22-days, we found that positive emotions accounted for the effect of awe on well-being.

All our findings were replicated across two samples, of community adults and healthcare professionals, with the exception of somatic health in Sample 2. In a day-to-day analysis of awe and somatic health, awe predicted fewer somatic health symptoms, but this effect did not hold when controlling for daily positive emotionality. However, our ancillary daily-level meta-analysis across both samples suggests that daily awe has a unique effect, beyond daily positive emotions, on stress, somatic health, and well-being. The unique effects of awe, beyond that of general positive emotions, in the within-person analyses but not in the between-person analyses suggest that daily experiences of awe are beneficial within the self but not in comparison to others, which might be more beneficial and consequential. Overall, these findings suggest that daily experiences of awe during times of chronic stress such as the COVID-19 pandemic in 2020 were beneficial for community adults and healthcare professionals alike.

This research builds on past work suggesting that awe is associated with reductions in stress and improvements in well-being^2, 3, 25, 29, 30^. Our findings also contribute to the literature by showing that daily experiences of awe are associated with reduced somatic health symptoms, such as body aches, insomnia, and fatigue. Our work provides support for the claim that awe serves as a pathway to physical and mental health^5^.

Future work can improve on the limitations of this investigation. Given our limited access to participants as reflected by our sample size, which was restricted by the pandemic, our samples are not very diverse. Future work can replicate this work with communities of color, for example, which have been severely impacted by the COVID-19 pandemic. Lastly, our measures were self-report and subjective in nature, future studies can build on our work and assess objective measures of health and examine whether experiences of awe can reduce the number of doctors’ visits, for example, or biomarkers of health and inflammation (e.g., interleukin-6)^28^.

As we deal with the consequences of the ongoing COVID-19 pandemic, it is ever more important to find means of cultivating awe—whether in the ordinary or the extraordinary. Experiences of awe whether out in nature, in daily narratives, or via nature videos in the lab have proved to be beneficial for mental and physical health. Our findings suggest that awe is a key ingredient for better health.

## Supplementary Information

Supplementary Information.

## Data Availability

The data associated with this manuscript are available upon reasonable request to the corresponding author.
