# Supplementary material for: The influences of daily experiences of awe on stress, somatic health, and well-being: a longitudinal study during COVID-19
Source: Sci Rep. 2023 Jun 8;13:9336. doi: 10.1038/s41598-023-35200-w (PMC10248986; doi:10.1038/s41598-023-35200-w)
Supplement: Supplementary file 1 — Supplementary Information. [file 41598_2023_35200_MOESM1_ESM.pdf]

**Supplemental Material**

# The Influences of Daily Experiences of Awe on Stress, Somatic Health, and Well-being: A Longitudinal Study During COVID-19

María Monroy,* Özge Uğurlu, Felicia Zerwas, Rebecca Corona, Dacher Keltner,

Jake Eagle, & Michael Amster

* Corresponding Author: mariamonroy@berkeley.edu

## Section 1. Procedures

### *Online Zoom Sessions*

All participants from Sample 1 and Sample 2 took part in four online zoom sessions. These sessions were 60-minutes long and began on day 1 at 12:15 pm PST for healthcare professionals and at 7 pm PST for community participants. The first session consisted of an overview of the study, an overview of the science of awe, and a discussion of how to find awe in daily life (by paying attention to the environment, slowing down and pausing, and expanding on those moments). The three additional zoom sessions were for participant support (on days 8, 15, and 22).

### *Planned Data Cleaning Procedure*

Given our analytic plan, a multilevel-modeling approach to examine within-person effects, and the complexity of some of the models, the final sample included participants with at least five

diary entries. Note that the more complex the model, the more random effects, and the more data it'll need per person. To ensure each diary entry was reflective of a specific day, entries submitted after 6 am the following day were also excluded from the final sample used for analyses. Similarly, because of time differences around the world, we also excluded participants outside the USA—international participants. The following are the sample sizes step by step for Sample 1 and Sample 2.

Community Sample:

1. 386 participants completed the entrance survey.
2. 357 completed the daily diary.
3. Exclusion criteria:
    a. Removed late entries between 6am and 4pm— *N* = 352
    b. Removed international participants— *N* = 344
    c. Removed those with less than 5 entries — *N* = 269 (Final Sample).

Healthcare Sample:

1. 227 participants completed the entrance survey.
2. 202 completed the daily diary.
3. Exclusion criteria:
    a. Removed late entries between 6am and 4pm— *N* = 199
    b. Removed international participants— *N* = 191
    c. Removed those with less than 5 entries— *N* = 145 (Final Sample).

## Section 2. Power Analyses

### ***Sample 1: Community Adults***

Given that there is no set standard on how to calculate power for multilevel models, we took two approaches. First, using G*Power (version 3.1.9.3; Faul et al., 2009), a post-hoc sensitivity analysis was conducted for a multiple regression, random model with a one-tailed test. Given our sample size (*N* = 269), alpha = 0.05, and a null hypothesis of 0, our study is powered to detect effects of .029 or larger at 80% power. However, given the nature of our data, diaries nested in participants, we conducted further sensitivity analysis for within-person effects. We used the *simr* package (version 1.0.6; Green & MacLeod, 2016) to simulate data based on our original

model data and estimate power from how many observed *t* values were significant. We used the awe and somatic health bivariate model because it had the smallest effect size (β = - 0.09) relative to the other models. To obtain standardized effects, we z-scored all variables across the entire sample. Figure S1 illustrates the distribution of power estimates using 1000 simulations for each potential β estimate. Based on the sensitivity curve, we estimate that we achieved 90% power to detect effects of approximately z-scored β = -.02 or larger. Note that these simulations are computationally intensive for our computing equipment to perform; thus, we selected a few points.

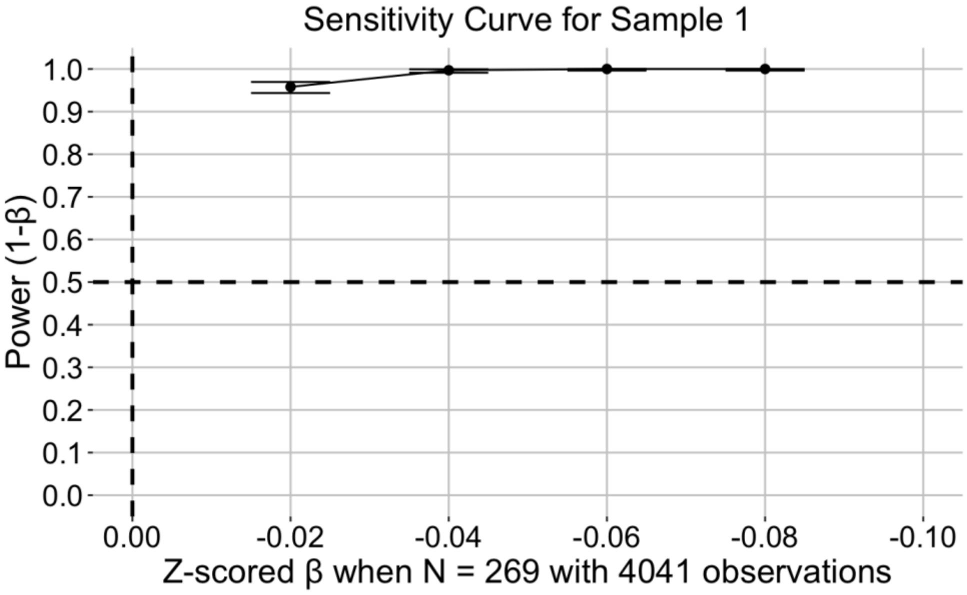


**Figure S1.** Sensitivity curve for sample of community adults.

***Sample 2: Healthcare Professionals***

For Sample 2, in a similar manner as Sample 1, we conducted a sensitivity analysis using the *simr* package (version 1.0.6; Green & MacLeod, 2016). As in Sample 1, we used the awe and somatic health bivariate model because it had the smallest effect size (β = - 0.11) relative to the

other models. To obtain standardized effects, we z-scored all variables across the entire sample. These simulations are computationally intensive for our computing equipment to perform; thus, we selected a few points. Figure S2 illustrates the distribution of power estimates using 1000 simulations for each potential β estimate. Based on the sensitivity curve, we estimate that we achieved 90% power to detect effects of approximately z-scored β = - .05 or larger.

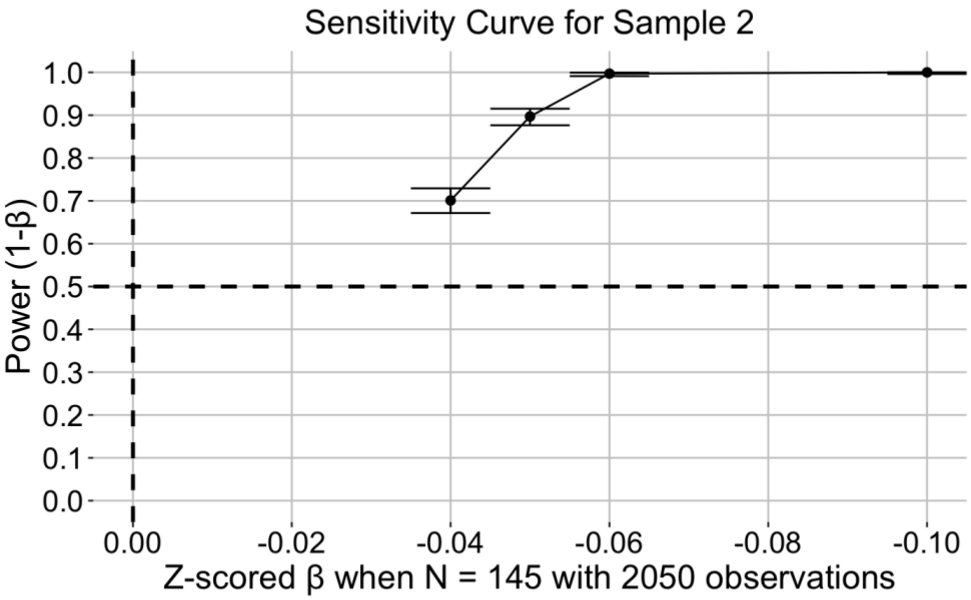


**Figure S2.** Sensitivity curve for sample of healthcare professionals.
